# Supplementary material for: First record of the complete mitochondrial genome of Streblote castanea (Swinhoe, 1892) (Lepidoptera: Lasiocampidae)
Source: Mitochondrial DNA B Resour. 2026 Feb 23;11(3):440–4. doi: 10.1080/23802359.2025.2609359 (PMC12931351; doi:10.1080/23802359.2025.2609359)
Supplement: Revised manuscript.docx [file TMDN_A_2609359_SM8704.docx]

First record of the complete mitochondrial genome of *Streblote castanea* (Swinhoe, 1892) (Lepidoptera: Lasiocampidae)

He-Wang Wang^a^, Xuan Zhou^a^, Fu-Ying Guo^a^, Hui-Ting Zhu^a^, Shi-Yuan Li^a^ and Xing Wang^[[1]](#footnote-1)^

^a^Qiongtai Normal University, Haikou, China

**Abstract**

This study reports the first complete mitochondrial genome (mitogenome) of *Streblote castanea* (Lepidoptera: Lasiocampidae), a keystone pest of tropical mangrove ecosystems. The mitogenome, spanning 15,400 bp, comprises 13 protein-coding genes (PCGs), 22 tRNA genes, two rRNA genes, and an A+T-rich control region, with a total AT content of 78.6%. This assembly addresses the critical genomic data gap for *Streblote* (Hübner, 1820) and Lasiocampidae, providing foundational insights for taxonomic revision, population dynamics tracing, and targeted biocontrol strategies in ecologically fragile mangrove habitats.

Keywords: Lappet moths; mangrove pest; mitogenome; phylogenetic trees

**Introduction**

The past decade has witnessed an exponential increase in insect genomic data availability, with mitochondrial (mt) genomes emerging as the most widely utilized molecular markers in systematic entomology due to their maternal inheritance, compact structure, and moderate evolutionary rates. Currently, publicly available insect mitochondrial genomes outnumber nuclear genomes by more than tenfold, and they cover the majority of insect orders (Cameron 2014). Against this backdrop, recent breakthroughs have been made in understanding the phylogenetic relationships among superfamilies of Lepidoptera: a study (Chen et al. 2025) based on large-scale genomic data strongly supports several new sister-group relationships. These findings provide a new phylogenetic framework for understanding the adaptive evolution of key lepidopteran lineages. However, the uniparental inheritance mode and limited genetic information of mitochondrial genomes may introduce biases in phylogenetic reconstruction (Bernt et al. 2013). Consequently, integrating mitochondrial and nuclear genomic data has become a critical approach for resolving insect evolutionary mechanisms (Kawahara et al. 2019), as this multi-omics strategy not only mitigates phylogenetic artifacts from single-marker analyses but also elucidates nuclear-mitochondrial coevolution during adaptive evolution (Hill 2016; Sloan et al. 2018). *Streblote castanea* (Swinhoe, 1892) is a keystone pest in tropical mangrove ecosystems. It is distributed in India, Sri Lanka, the Philippines, and Hainan (China) (Holloway 1987; Liu and Wu 2006; Prozorov et al. 2022). The larvae are leaf-feeders that engage in binge-feeding, displaying a body coloration that closely resembles that of the surrounding branches and trunks. They exhibit high levels of concealment, robust activity, and considerable migration capacity, and they have a prolonged larval stage (≥40 days). The mature larvae of this species form cocoons on the trunks of trees and undergo a brief pupal period. Adults exhibit high fertility levels and produce a substantial number of eggs (Ong et al. 2010). It causes severe ecological disruption by defoliating seedlings of the dominant mangrove species *Lumnitzera racemosa* (Willd). Despite its ecological significance, research has predominantly focused on morphological descriptions and taxonomic revisions of geographic populations (Prozorov et al. 2022), with molecular investigations remaining critically underdeveloped. As of 2024, neither mitochondrial nor nuclear genome sequences of this species are available in the NCBI database, and the molecular data void for the genus *Streblote* has hindered cross-generic comparative evolutionary analyses. This genomic gap is particularly pronounced within the family Lasiocampidae. This study presents the first complete mitochondrial genome assembly and annotation of *S. castanea*, accompanied by molecular phylogenetic reconstruction. Our findings advance the understanding of mitogenomic architecture in this ecologically destructive species, providing a foundation for taxonomic refinement, population dispersal tracing, and targeted biocontrol strategies. Additionally, this work clarifies the phylogenetic position of *Streblote castanea* within Lasiocampidae and contributes a pivotal case study for investigating nuclear-mitochondrial coevolutionary dynamics.

| 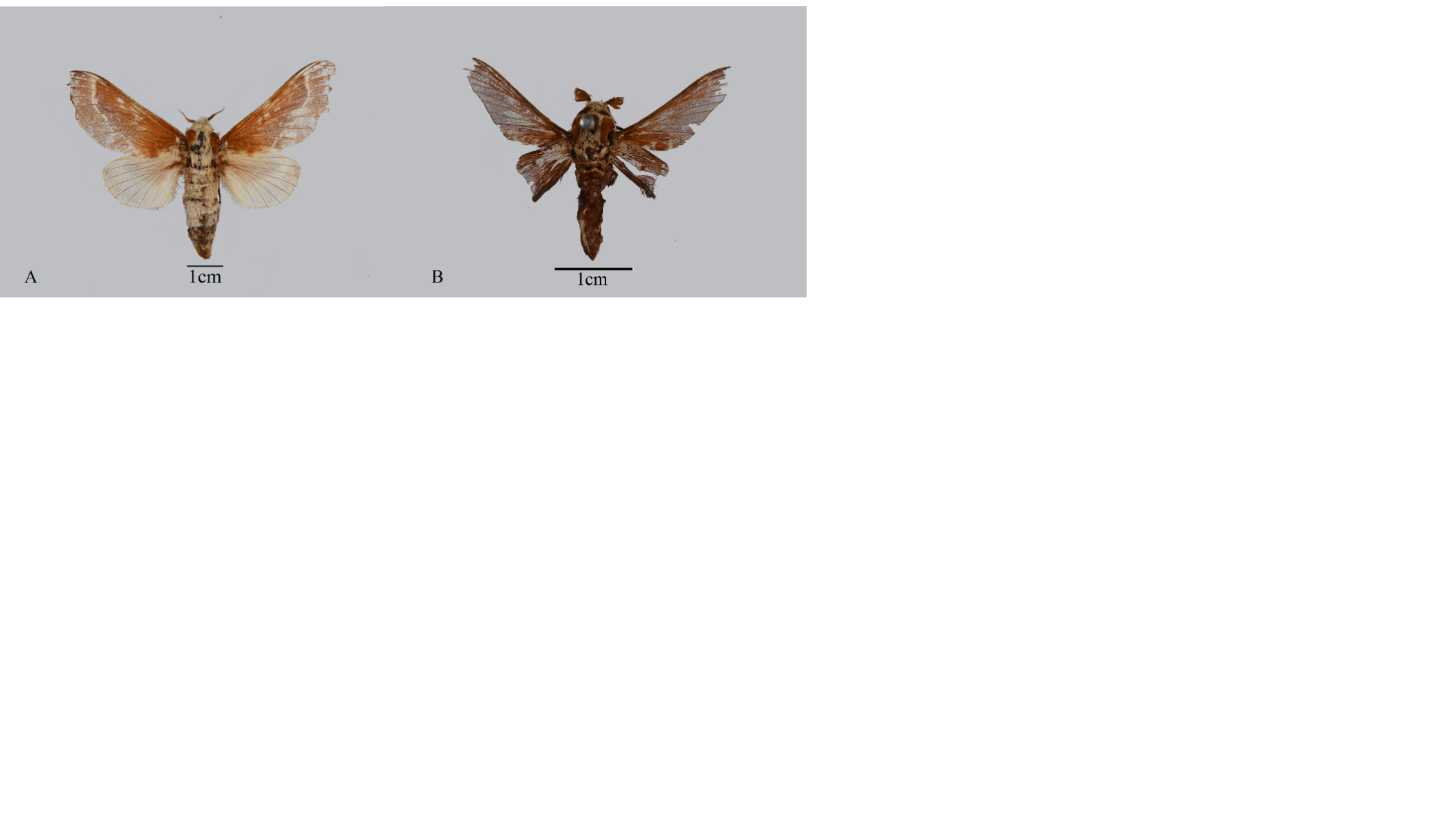 |
| --- |

Figure 1. *Streblote castanea* (Swinhoe, 1892) adults: (A) female; (B) male. Photo taken by the first author (Study used larval samples).

**Materials and Methods**

On September 26, 2023, Xing Wang collected *S. castanea* (Figure 1) cocoons through field collection at the Sanya Tiefang Mangrove Nature Reserve (18°15-18°17′N, 109°42′- 109°44′E, Linwang, Linwang Town, Sanya City, China). Meanwhile, the specimens and genomic DNA were deposited in the Insect Herbarium of Qiongtai Normal University (contact person: Xing Wang, email: xingwanghjt@163.com), Haikou City, Hainan Province, China under the voucher number S-c01. Genomic DNA was extracted from larval body tissue using the SteadyPure Universal Genomic DNA Extraction Kit Ver.1.0 protocol and sequenced on the Illumina NovaSeq 6000 platform (2×150 bp) at Berry Genomics (Beijing, China). Raw reads were filtered with FASTP v0.23.2 (Chen et al. 2018), and de novo mitochondrial genome assembly was subsequently performed using NOVOPlasty v4.3.1 (Dierckxsens et al. 2017) and GetOrganelle v1.7.6 (Jin et al. 2020). The mitogenome was annotated via the MITOS web server (Bernt et al. 2013), and annotation results were subsequently validated by NCBI BLAST to confirm gene boundaries and identities, with tRNA genes verified by tRNAscan-SE (Lowe and Chan 2016) and manually refined in Geneious Prime v10.1.4 (Kearse et al. 2012). Protein-coding genes (PCGs) and rRNAs were identified through comparative alignment with reference mitogenomes. A circular genome map was generated using Proksee (Grant et al. 2023). For phylogenetic reconstruction, 13 PCGs and 2 rRNAs from 16 mitogenomes (9 Lasiocampidae, 5 Noctuidae, 1 Crambidae, 1 Sphingidae) were analyzed. The GenBank accession numbers are listed as follows: *Nomophila noctuella* (Denis and Schiffermüller, 1775) NC_025764 (Tang.et al. 2014), *Ischyja manlia* (Cramer, 1766) NC_065824 (Riyaz et al. 2022), *Xanthodes intersepta* Guenée, 1852 NC_062099 (Liang et al. 2022), *Helicoverpa assulta* (Guenée, 1852) MZ618264.1 (Liang et al. 2021), *Spodoptera depravata* (Butler, 1879) NC_061562 (Liang et al. 2022), *Condica illecta* (Walker, 1865) MW768082 (Li et al. 2021), *Euthrix laeta* (Walker, 1855) NC_031507 (Wu et al. 2016), *Apatelopteryx phenax* de Joannis, 1912 KJ508055 (Timmermans et al. 2014), *Streblote castanea* PV275244 (present study), *Dendrolimus kikuchii* Matsumura, 1927 MF100138 (Chen et al. 2017), *Dendrolimus houi* Lajonquière, 1973 NC_039840 (Qin et al. 2018), *Dendrolimus spectabilis* (Butler, 1877) NC_025763 (Tang et al. 2014), *Dendrolimus tabulaeformis* Tsai et Liu, 1962 NC_027157 (Qin et al. 2015), *Dendrolimus punctatus* (Walker, 1855) MN605220 (Du. 2019), *Dendrolimus superans* (Butler, 1877) KY000414 (Qin et al. 2016), *Marumba cristata* (Butler, 1875) OP359030 (Zheng, 2022),. Amino acid sequences were aligned with MAFFT v7.149 (Katoh and Standley 2013), concatenated using FASConCAT-g v1.05.1 (Kück and Longo 2014), and partitioned under optimal models selected by ModelFinder v2.1.1 (Lanfear et al. 2017). Maximum-Likelihood analysis was performed using IQ-TREE v2 (Minh et al. 2020) with 1000 bootstrap replications, and Sphingidae as an outgroup. Phylogenetic analysis was conducted under the best-fit model GTR+F+I+G4, which was selected via the ModelFinder program integrated in IQ-TREE. Bayesian inference was completed using PhyloSuite v1.2.2. The final phylogenetic tree was visualized using
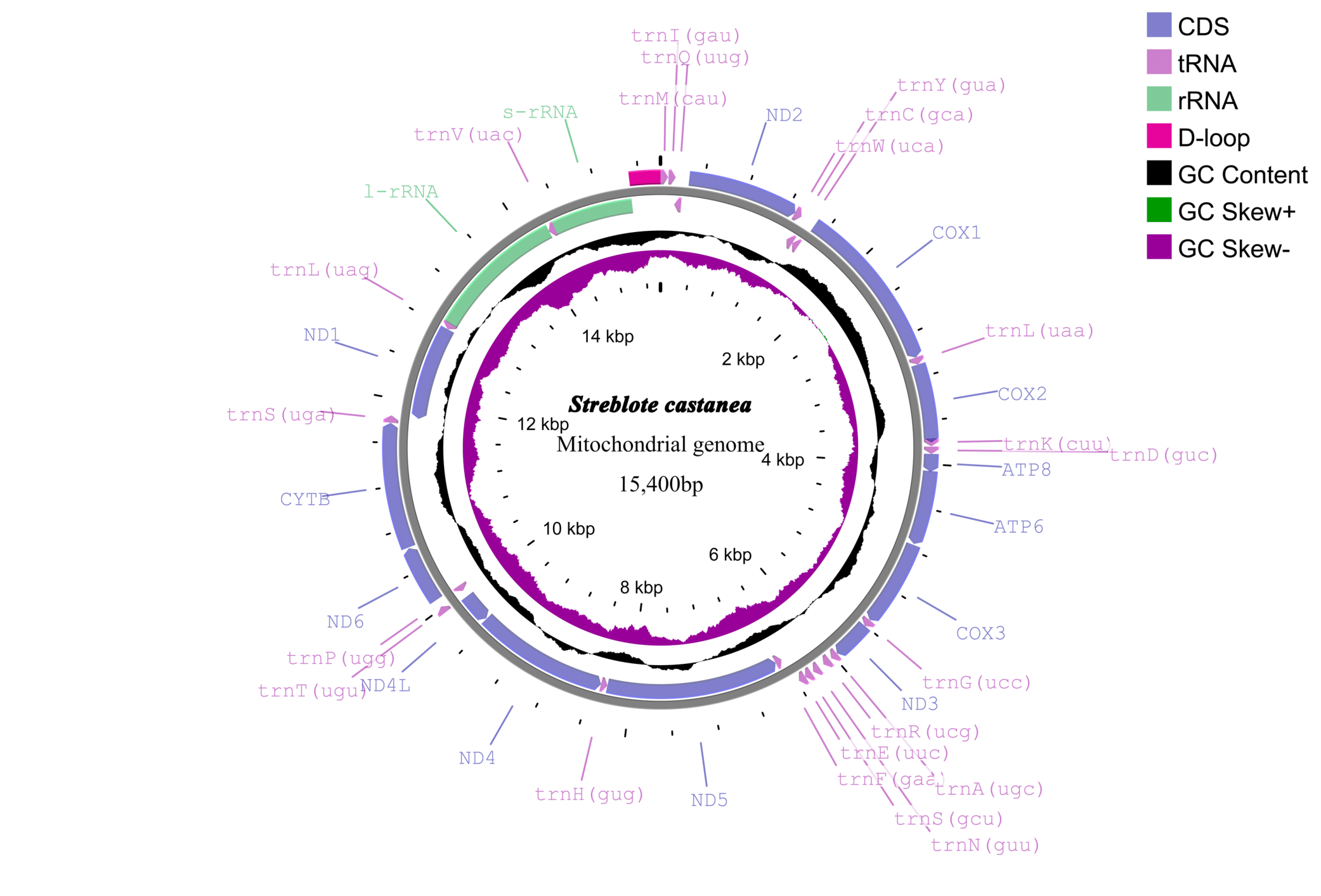
FigTree v1.4.4, which is distributed under the GNU General Public License v2 (GPLv2).

Figure 2. Diagram of the mitotic genome pattern of *Streblote castanea*. Pink arrows indicate the direction of gene transcription. Genes outside the circle are encoded by the majority strand (J-strand); genes inside the circle are encoded by the minority strand (N-strand).

**Results**

The full mitogenome of *S. castanea* (GenBank accession number: PV275244) is 15400 bp in size, with an average coverage depth of 227.2× (Figure S1). The mitogenome has a non-coding control region (A + T rich region, D-loop), along with 13 protein-coding genes (PCGs), 22 transfer RNA (tRNA) genes, and 2 ribosomal RNA (rRNA) genes. The nucleotides consist of A, C, G, and T, accounting for 39.5%, 13.5%, 7.9%, and 39.1%, respectively. The AT nucleotide content is 78.6% (Figure 2). Out of the 37 genes, 23 are located on the J-strand, including nine PCGs (*ND2, ND3, ND6, COX1, COX2, COX3, ATP6, ATP8, CYTB*) and 14 tRNAs (*trnM, trnI, trnW, trnL^UAA^, trnK, trnD, trnG, trnA, trnR, trnN, trnS^GCU^, trnE, trnT, trnS^UGA^*). The N-strand harbors the remaining 4 PCGs (*ND1, ND4, ND4L, ND5*), 8 tRNAs, and both rRNA genes (rrnS and rrnL), alongside the AT-rich control region. The 12 PCGs start with typical ATN initiation codons (three with ATT, two with ATA, one with ATC and six with ATG), except for COX1 which begins with CGA. All of the PCGs end with the typical stop codons except for COX1 and ND4 which use T as an incomplete termination codon. The mitochondrial genome annotation revealed 22 transfer RNA (tRNA) genes exhibiting a size range of 65 bp (*trnP*) to 71 bp (*trnK*). The small ribosomal RNA (rrnS) and large ribosomal RNA (rrnL) subunits were as 815 bp and 1,377 bp in length, respectively. In our study, 13 PCGs of 16 species were used in phylogenetic analysis.


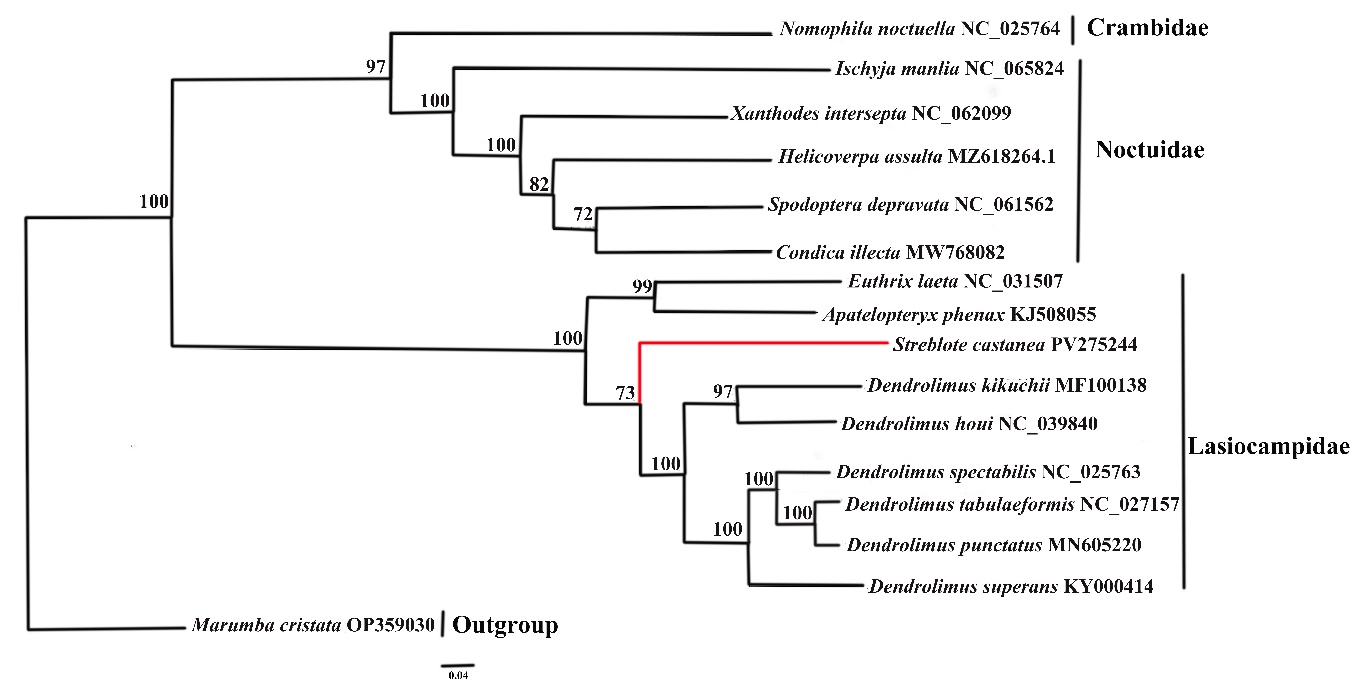


Figure 3. The maximum-likelihood (ML) phylogenetic tree of Lepidoptera species based on complete mitochondrial genomes. All species involved in the tree have scientific names with accession numbers on the right side. GenBank accession numbers are shown in Table S1. The tree was inferred using IQ-TREE under the GTR+F+I+G4 model. Nodal support was evaluated from 1000 bootstrap replications, with values shown at the nodes. Marumba cristata (Sphingidae) was designated as the outgroup. The tree topology was visualized using FigTree v1.4.4.

**Discussion and Conclusions**

Here, we report the assembly and annotation of the complete mitochondrial genome for *S. castanea*, adding a new genomic resource for the family Lasiocampidae. The genome (15,400 bp) is within the expected 15–16 kb range for Lepidoptera and shows high structural similarity to related species like *Dendrolimus punctatus* and *Euthrix laeta* (Wu et al. 2016; Du 2019). Both gene order and orientation match the ancestral arrangement found across the superfamily Lasiocampoidea. This supports the view that insect mitochondrial architectures remain evolutionarily conserved (Qin et al. 2015; Chen et al. 2017). Additionally, the high A+T bias (78.6%) in *S. castanea* aligns with typical lepidopteran patterns, likely resulting from the bias of mitochondrial DNA polymerase during replication (Cameron 2014).

In our phylogenetic analysis of 13 PCGs, *S. castanea* appears as a sister group to the genus *Dendrolimus* (Figure 3). This molecular evidence corroborates traditional morphological classifications that place these genera together in the subfamily Lasiocampinae (Holloway 1987; Prozorov et al. 2022). However, nodal support varied between methods. While Bayesian Inference (BI) strongly supported this sister-group relationship, the bootstrap support in Maximum Likelihood (ML) analysis was moderate. Such incongruence is not rare in studies of rapidly radiating lineages and may stem from saturation of substitution sites or long-branch attraction (Cameron 2014). Given the high posterior probability in BI (Figure S3) and the morphological context, we consider the *Streblote-Dendrolimus* relationship to be robust.

Overall, this study establishes the first mitogenome for *S. castanea*, confirming its typical genomic features and clarifying its taxonomy. These results contribute to our understanding of Lasiocampidae evolution. While mitochondrial data provides key insights, the variation in branch support suggests that future research should include nuclear markers or transcriptomic data (Chen et al. 2025). This approach will be essential to fully resolve complex species-level relationships and overcome the limits of single-locus phylogenies.

**Authors’ contributions**

Writing the paper, uploading the sequencing data and analyzing the data: WHW. Revising and checking the paper: WX. Collecting samples of *S. castanea*: WX. Taking photographs and preparing the specimens for the experiments: WHW, ZX, ZHT, GFY, and LSY. Identifying the insects and providing the necessary equipment for the experiments: WX. All authors agree to be responsible for all aspects of the work. All authors agree to be responsible for all aspects of the work.

**Ethics statement**

The focal species used in this study are not protected by the Convention on International Trade in Endangered Species of Wild Fauna and Flora (CITES) and the Wildlife Act, and their status has not been assessed by the World Conservation Union (IUCN). Specimens were collected and handled in accordance with the Regulations on the Management of Laboratory Animals in Hainan Province.

**Disclosure statement**

The authors disclaim any conflict of interest and take responsibility for the content.
**Funding**

This work was financially assisted by the National Natural Science Foundation of China [32360134] and the Key Project of Qiongtai Normal University [qtky202402].

**ORCID**

Wang-He Wang iD: <https://orcid.org/0009-0006-8233-4160>

Xing Wang iD: <http://orcid.org/0000-0001-6815-5912>

Xuan Zhou iD: <https://orcid.org/0009-0008-9546-4620>

Fu-Ying Guo iD: <https://orcid.org/0009-0007-2497-1791>

Hui-Ting Zhu iD: <https://orcid.org/0009-0007-2684-493X>

Shi-Yuan Li iD: <https://orcid.org/0009-0007-1745-8201>

**Data availability statement**

The genome sequence data supporting the results of this study are openly available in GenBank at NCBI under accession no. PV275244. The associated BioProject, BioSample, and SRA numbers are PRJNA1260701, SAMN48402020, and SRR33481764, respectively.

**References**

Bernt M, Donath A, Jühling F, Externbrink F, Florentz C, Fritzsch G, Pütz J, Middendorf M, Stadler PF. 2013. MITOS: improved de novo metazoan mitochondrial genome annotation. Mol Phylogenet Evol. 69(2): 313-319. doi:[10.1016/j.ympev.2012.08.023.](https://pubmed.ncbi.nlm.nih.gov/22982435/)

Cameron SL. 2014. Insect mitochondrial genomics: implications for evolution and phylogeny. Annu Rev Entomol. 59: 95-117. doi:[10.1146/annurev-ento-011613-162007.](https://pubmed.ncbi.nlm.nih.gov/24160435/)

Chen Q, Deng M, Dai X, Wang W, Wang X, Chen SL, Huang GH. 2025. Phylogenomic data exploration with increased sampling provides new insights into the higher-level relationships of butterflies and moths (Lepidoptera). J Syst Evol. 63(5): 1133-1153. doi:<https://doi.org/10.1111/jse.13180>.

Chen S, Zhou Y, Chen Y, Gu J. 2018. Fastp: an ultra-fast all-in-one FASTQ preprocessor. Bioinformatics. 34(17): i884-i890. doi:[10.1093/bioinformatics/bty560.](https://pubmed.ncbi.nlm.nih.gov/30423086/)

Chen Z, Xu C, Ouyang Y. 2017. NCBI GenBank. https://www.ncbi.nlm.nih.gov/nuccore/MF100138.

Dierckxsens N, Mardulyn P, Smits G. 2017. NOVOPlasty: de novo assembly of organelle genomes from whole genome data. Nucleic Acids Res. 45(4): e18. doi:[10.1093/nar/gkw955.](https://pubmed.ncbi.nlm.nih.gov/28204566/)

Du H. 2019. NCBI GenBank. https://www.ncbi.nlm.nih.gov/nuccore/MN605220.

Grant JR, Enns E, Marinier E, Mandal A, Herman EK, Chen C, Graham M, Van Domselaar G, Stothard P. 2023. Proksee: in-depth characterization and visualization of bacterial genomes. Nucleic Acids Res. 51(W1): W484-W492. doi:[10.1093/nar/gkad326.](https://academic.oup.com/nar/article/51/W1/W484/7151341)

Hill GE. 2016. Mitonuclear coevolution as the genesis of speciation and the mitochondrial DNA barcode gap. Ecol Evol. 6: 5831-5842. doi: [10.1002/ece3.2338.](https://onlinelibrary.wiley.com/doi/10.1002/ece3.2338)

Holloway JD, 1987. The moths of Borneo: Superfamily Bombycoidea: families Lasiocampidae, Eupterotidae, Bombycidae, Brahmaeidae, Saturniidae, Sphingidae. Kuala Lumpur: Southdene. 199 pp.

Jin JJ, Yu WB, Yang JB, Song Y, DePamphilis CW, Yi TS, Li DZ. 2020. GetOrganelle: a fast and versatile toolkit for accurate de novo assembly of organelle genomes. Genome Biol. 21(1): 241. doi: [10.1186/s13059-020-02154-5.](https://genomebiology.biomedcentral.com/articles/10.1186/s13059-020-02154-5)

Katoh K, Standley DM. 2013. MAFFT multiple sequence alignment software version 7: improvements in performance and usability. Mol Biol Evol. 30(4): 772-780. doi:[10.1093/molbev/mst010.](https://pubmed.ncbi.nlm.nih.gov/23329690/)

Kawahara AY, Plotkin D, Espeland M, Meusemann K, Toussaint EFA, Donath A, Gimnich F, Frandsen PB, Zwick A, dos Reis M, Barberh JR, Peters RS, Liu S, Zhou X, Mayer C, Podsiadlowski L, Storer C, Yack JE, Misof B, Breinholt JW. 2019. Phylogenomics reveals the evolutionary timing and pattern of butterflies and moths. Proc Natl Acad Sci USA. 116(45): 22657-22663. doi: 10.1073/pnas.1907847116.

Kearse M, Moir R, Wilson A, Stones-Havas S, Cheung M, Sturrock S, Buxton S, Cooper A, Markowitz S, Duran C. 2012. Geneious Basic: an integrated and extendable desktop software platform for the organization and analysis of sequence data. Bioinformatics. 28(12): 1647-1649. doi: [10.1093/bioinformatics/bts199.](https://pubmed.ncbi.nlm.nih.gov/22543367/)

Kück P, Longo GC. 2014. FASconCAT-G: extensive functions for multiple sequence alignment preparations concerning phylogenetic studies. Front Zool. 11(1): 81. doi: [10.1186/s12983-014-0081-x.](https://frontiersinzoology.biomedcentral.com/articles/10.1186/s12983-014-0081-x)

Lanfear R, Frandsen PB, Wright AM, Senfeld T, Calcott B. 2017. Partition Finder 2: new methods for selecting partitioned models of evolution for molecular and morphological phylogenetic analyses. Mol Biol Evol. 34(3): 772-773. doi: [10.1093/molbev/msw260.](https://academic.oup.com/mbe/article/34/3/772/2738784)

Liang X, Zhang L, Xiao Y. 2021. NCBI GenBank. <https://www.ncbi.nlm.nih.gov/nuccore/MZ618264.1>.

Liang X, Zhang L, Xiao Y. 2022. NCBI GenBank. <https://www.ncbi.nlm.nih.gov/nuccore/NC_061562>.

Liang X, Zhang L, Xiao Y. 2022. NCBI GenBank. <https://www.ncbi.nlm.nih.gov/nuccore/NC_062099>.

Li Q, Feng X, Wu H, Wang G, Zheng Y. 2021. NCBI GenBank. <https://www.ncbi.nlm.nih.gov/nuccore/MW768082>.

Liu YQ, Wu CS, 2006. Zoology of China: Insects. Volume 47. BeiJing (China): Science Publishing House.

Lowe TM, Chan PP. 2016. tRNAscan-SE On-line: integrating search and context for analysis of transfer RNA genes. Nucleic Acids Res. 44(W1): W54-W57. doi: [10.1093/nar/gkw413.](https://academic.oup.com/nar/article/44/W1/W54/2499377?login=false)

Minh BQ, Schmidt HA, Chernomor O, Schrempf D, Woodhams MD, Von Haeseler A, Lanfear R. 2020. IQ-TREE 2: new models and efficient methods for phylogenetic inference in the genomic era. Mol Biol Evol. 37(5): 1530-1534. doi: [10.1093/molbev/msaa015.](https://pubmed.ncbi.nlm.nih.gov/32011700/)

Ong SP, Cheng S, Chong VC, Tan YS. 2010. Pests of Planted Mangroves in Peninsular Malaysia. Kuala Lumpur (Malaysia): Forest Research Institute Malaysia.Prozorov AM, Prozorova TA, Spitsyn VM, Spitsyna EA, Volkova JS, Yakovlev RV, Meier J, Saldaitis A, Revay EE and Müller GC. 2022. Notes on *Streblote* (Lepidoptera, Lasiocampidae, Lasiocampinae) from the Malay Archipelago with two new species description. Ecol Montenegrina. 58(15): 14-28. doi: [10.37828/em.2022.58.2.](https://cris.haifa.ac.il/en/publications/notes-on-streblote-lepidoptera-lasiocampidae-lasiocampinae-from-t)

Qin J, Zhang AB. 2015. NCBI GenBank. <https://www.ncbi.nlm.nih.gov/nuccore/NC_027157>.

Qin J, Zhang AB. 2016. NCBI GenBank. <https://www.ncbi.nlm.nih.gov/nuccore/KY000414>.

Qin J, Zhang AB. 2018. NCBI GenBank. <https://www.ncbi.nlm.nih.gov/nuccore/NC_039840>.

Riyaz M, Shah RA, Savarimuthu I, Kuppusamy S. 2022. NCBI GenBank. <https://www.ncbi.nlm.nih.gov/nuccore/NC_065824>.

Sloan DB, Warren JM, Williams AM. 2018. Cytonuclear integration and co-evolution. Nat Rev Genet. 19(10): 635-648. doi: [10.1038/s41576-018-0035-9.](https://www.nature.com/articles/s41576-018-0035-9)

Tang M, Tan M, Meng G, Yang S, Su X, Liu S, Song W, Li Y, Wu Q, Zhang A, Zhou X. 2014. NCBI GenBank. <https://www.ncbi.nlm.nih.gov/nuccore/NC_025763>.

Tang M, Tan M, Meng G, Yang S, Su X, Liu S, Song W, Li Y, Wu Q, Zhang A, Zhou X. 2014. NCBI GenBank. <https://www.ncbi.nlm.nih.gov/nuccore/NC_025764>.

Timmermans MJ, Lees D, Simonsen T. 2014. NCBI GenBank. <https://www.ncbi.nlm.nih.gov/nuccore/KJ508055>.

Wu L, Wu X, Xiong X, Xin T, Wang Y, Zou Z, Xia B. 2016. NCBI GenBank. <https://www.ncbi.nlm.nih.gov/nuccore/NC_031507>.

Zheng X. 2022. NCBI GenBank. <https://www.ncbi.nlm.nih.gov/nuccore/OP359030>.

Supplemental material

Figure S1. The coverage depth of the mitochondrial genome of *Streblote castanea.*


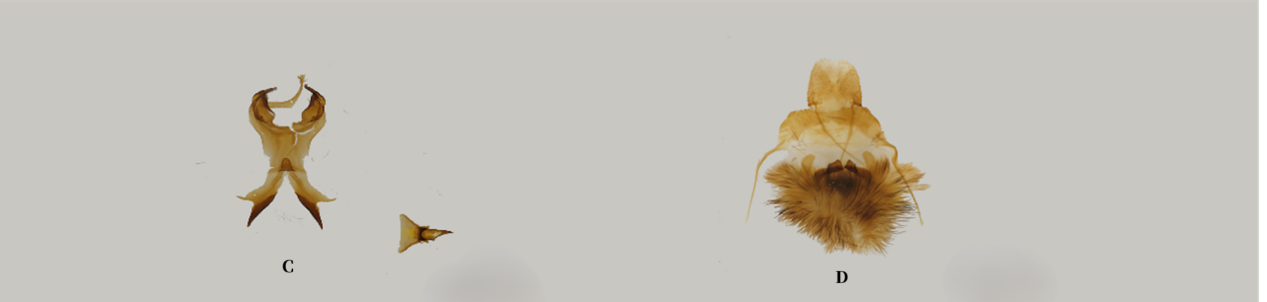
Figure S2. *Streblote castanea* (Swinhoe, 1892): (C) male genitalia (right side is aedeagus); (D) female genitalia. Photo by the first author.

| Family | Species | Accession Number | Reference |
| --- | --- | --- | --- |
| Crambidae | *Nomophila noctuella* | NC_025764 | Tang et al. 2014 |
| Noctuidae | *Ischyja manlia* | NC_065824 | Riyaz et al. 2022 |
| Noctuidae | *Xanthodes intersepta* | NC_062099 | Liang et al. 2022 |
| Noctuidae | *Helicoverpa assulta* | MZ618264.1 | Liang et al. 2021 |
| Noctuidae | *Spodoptera depravata* | NC_061562 | Liang et al. 2022 |
| Noctuidae | *Condica illecta* | MW768082 | Li et al. 2021 |
| Lasiocampidae | *Euthrix laeta* | NC_031507 | Wu et al. 2016 |
| Lasiocampidae | *Apatelopteryx phenax* | KJ508055 | Timmermans et al. 2014 |
| Lasiocampidae | *Streblote castanea* | PV275244 | Present study |
| Lasiocampidae | *Dendrolimus kikuchii* | MF100138 | Chen et al. 2017 |
| Lasiocampidae | *Dendrolimus houi* | NC_039840 | Qin et al. 2018 |
| Lasiocampidae | *Dendrolimus spectabilis* | NC_025763 | Tang et al. 2014 |
| Lasiocampidae | *Dendrolimus tabulaeformis* | NC_027157 | Qin et al. 2015 |
| Lasiocampidae | *Dendrolimus punctatus* | MN605220 | Du 2019 |
| Lasiocampidae | *Dendrolimus superans* | KY000414 | Qin et al. 2016 |
| Sphingidae | *Marumba cristata* | OP359030 | Zheng 2022 |

Table S1. List of species and GenBank accession numbers used for phylogenetic analysis in this study.


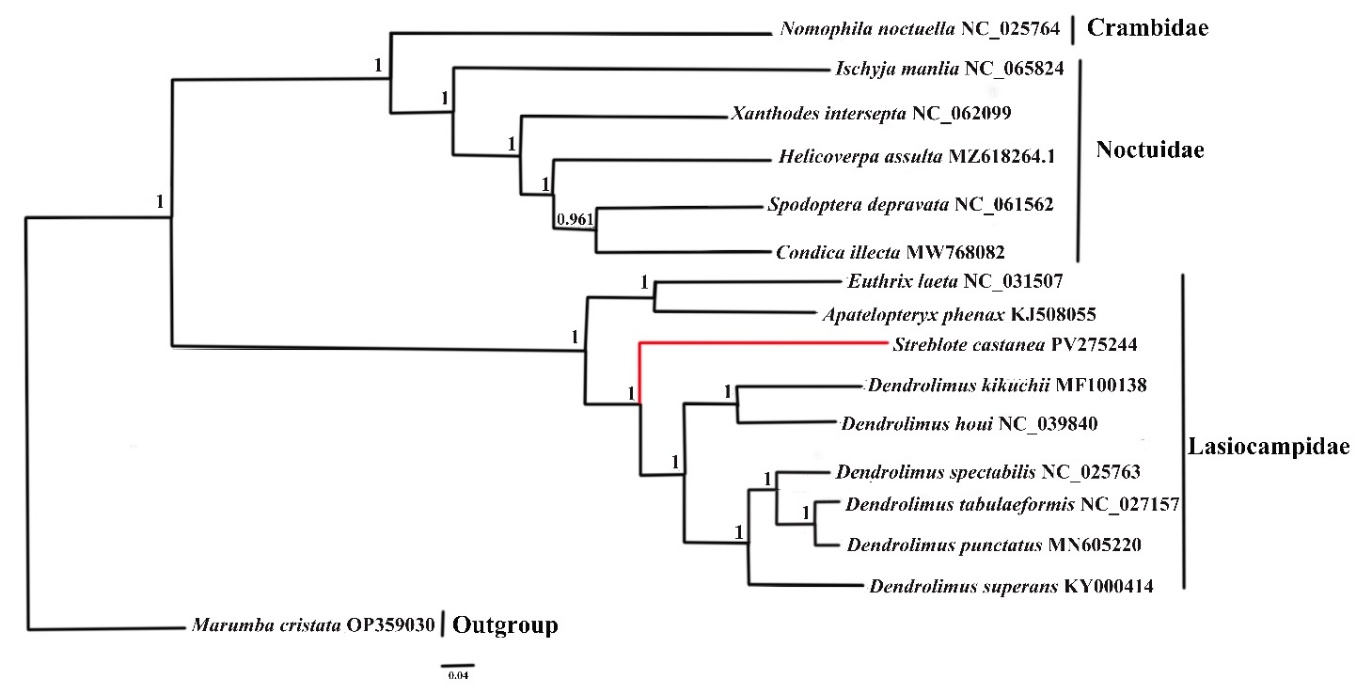


Figure S3 Phylogenetic tree of the Lepidoptera species (BI).

1. **CONTACT: Xing Wang, E-mail:** [**xingwanghjt@163.com**](mailto:xingwanghjt@163.com)**, Qiongtai Normal University, Haikou, China.**  [↑](#footnote-ref-1)
